# Supplementary material for: An adaptive variational algorithm for exact molecular simulations on a quantum computer
Source: Nat Commun. 2019 Jul 8;10:3007. doi: 10.1038/s41467-019-10988-2 (PMC6614426; doi:10.1038/s41467-019-10988-2)
Supplement: Supplementary file 1 — Supplementary Information [file 41467_2019_10988_MOESM1_ESM.pdf]

Supplementary Information for: An adaptive variational algorithm for exact molecular simulations on a quantum computer

Grimsley et al.

I. SUPPLEMENTARY DISCUSSION

A. Derivation of ADAPT-VQE as a sparse-update FCI ansatz

In addition to the intuitive definition of the algorithm presented above, one can also view it as a version of full CI (FCI) VQE (using the ansatz definition in Eq. (7) of the main text), but with a sparsity constraint on the gradient-based update step, such that only single parameter updates are allowed per iteration. We will adopt a slightly more generic notation at this point to simplify the discussion:

$$\left|\psi^{\text{FCI}}\right\rangle = \prod_{k}^{\infty}\prod_{n}^{N^4} e^{\theta_n^k \hat{A}_n}\left|\psi^{\text{HF}}\right\rangle, \tag{1}$$

where $\hat{A}_n \in \left\{\hat{a}_p^\dagger \hat{a}_q - \hat{a}_q^\dagger \hat{a}_p, \hat{a}_p^\dagger \hat{a}_q^\dagger \hat{a}_r \hat{a}_s - \hat{a}_s^\dagger \hat{a}_r^\dagger \hat{a}_q \hat{a}_p\right\}$. The goal is to find parameters θ_m^k such that the energy functional is minimized to a value that is as close as possible to the FCI energy:

$$\frac{\partial}{\partial \theta_m^k}\left\langle\psi^{\text{HF}}\right| e^{-\theta_1^1 \hat{A}_1} e^{-\theta_2^1 \hat{A}_2} \cdots \hat{H} \cdots e^{\theta_2^1 \hat{A}_2} e^{\theta_1^1 \hat{A}_1}\left|\psi^{\text{HF}}\right\rangle = 0. \tag{2}$$

B. First iteration

If we initialize all of the parameters, θ_m^k, to zero, then the expectation value of the 0th iteration state, $\left|\psi^{(0)}\right\rangle$, would simply return the uncorrelated HF energy:

$$\left|\psi^{(0)}\right\rangle = \left|\psi^{\text{HF}}\right\rangle. \tag{3}$$

A gradient descent step from the HF state could then provide an improved set of parameters, lowering the expected energy. The gradient at the 0th iteration is quite simple due to the fact that all the θ_m^k vanish:

$$\frac{\partial E^{(0)}}{\partial \theta_m^k} = \frac{\partial}{\partial \theta_m^k}\left\langle\psi^{\text{HF}}\right| e^{-\theta_1^1 \hat{A}_1} e^{-\theta_2^1 \hat{A}_2} \cdots \hat{H} \cdots e^{\theta_2^1 \hat{A}_2} e^{\theta_1^1 \hat{A}_1}\left|\psi^{\text{HF}}\right\rangle\Big|_{\theta_m^k=0} = \left\langle\psi^{\text{HF}}\right| [H, \hat{A}_m] \left|\psi^{\text{HF}}\right\rangle. \tag{4}$$

Using the gradient to update all the parameters in the wavefunction would immediately lead to an exponentially complicated wavefunction which could no longer be simulated either classically or quantum mechanically, because each of the terms that were initially set to zero become non-zero, meaning that each term now has to appear in the circuit. The reason is that all k replicas have identical gradients:

$$\frac{\partial E^{(0)}}{\partial \theta_m^k} = \frac{\partial E^{(0)}}{\partial \theta_m^j} \text{for all } k, j. \tag{5}$$

This would mean that the ansatz would have an exponentially large number of terms after the first iteration, and also the circuit depth would be exponentially large. Alternatively, we choose to impose a sparsity constraint on the parameter update, such that only a single parameter may be updated at a time. This has the consequence that after

*Electronic address: nmayhall@vt.edu

Iteration	Finite Differences, s	Analytical, s	x Speedup
1	379.0	2.6	145.7
2	405.2	3.1	130.5
3	375.7	2.7	140.4
4	377.6	2.8	134.1
5	369.7	2.6	144.7
6	383.2	2.5	150.6
7	374.6	3.0	126.9
8	369.0	2.6	141.3
9	378.0	3.1	122.7
10	397.4	3.4	118.5
Average	380.9	2.8	134.6

Supplementary Table I: Timing comparison between finite difference and analytical gradients for several VQE iterations. System being studied is H_2O with STO-3G basis set. Ansatz has 140 parameters.

updating the single chosen parameter, the ansatz has only increased in complexity by a single non-zero parameter, and the resulting circuit has only grown in depth to accommodate the single extra one- or two-body operator:

$$\left|\psi^{(1)}\right\rangle = e^{\theta_p \hat{A}_p} \left|\psi^{\mathrm{HF}}\right\rangle . \tag{6}$$

To recover the greatest amount of electron correlation with the maximally compact wavefunction, we choose to update only the operator with the largest gradient. As a result of this update, the ansatz has grown from HF to a simple UCC-type ansatz with only a single excitation operator.

To determine the stepsize for this iteration (essentially a line-search), we perform a variational minimization via VQE:

$$E^{(1)} = \min_{\theta_p^1} \left\langle \psi^{\mathrm{HF}} \right| e^{-\theta_p^1 \hat{A}_p} \hat{H} e^{\theta_p^1 \hat{A}_p} \left|\psi^{\mathrm{HF}}\right\rangle . \tag{7}$$

The result of this relatively simple VQE experiment then provides the first iteration energy and wavefunction.

C. Second iteration

In the second iteration, the computation of the gradient of all the one- and two-body operators is required once again, and can be performed in a NISQ-friendly parallel execution on uncoupled quantum computers:

$$\frac{\partial E^{(1)}}{\partial \theta_m^k} = \langle \psi^{(1)} | [H, \hat{A}_m] | \psi^{(1)} \rangle . \tag{8}$$

However, because θ_p^1 has already converged in the previous VQE step, its gradient is zero. Just as in the previous iteration, a sparse update is performed to identify and insert only the operator with the largest magnitude gradient. This grows the ansatz by one parameter, θ_q^1. The second iteration energy is then obtained by a new VQE simulation for a UCC ansatz containing two excitation operators:

$$E^{(2)} = \min_{\theta_p^1, \theta_q^1} \left\langle \psi^{\mathrm{HF}} \right| e^{-\theta_p^1 \hat{A}_p} e^{-\theta_q^1 \hat{A}_q} \hat{H} e^{\theta_q^1 \hat{A}_q} e^{\theta_p^1 \hat{A}_p} \left|\psi^{\mathrm{HF}}\right\rangle . \tag{9}$$

We emphasize that it is not only the new parameter which is optimized, but all previously added parameters are updated as well. Future iterations follow similarly.

D. Analytic Gradients

This section presents a derivation of the analytic gradient method used to improve both the computational efficiency and the numerical precision of the classical simulation of the parameter re-optimization that occurs in step 6 of the

ADAPT-VQE algorithm, as described in the main text. It should be noted, however, that the gradient algorithm we present here is in fact completely general and could be used for an arbitrary Trotterized ansatz, not just ADAPT.

Consider a general Trotterized ansatz, $|\phi\rangle$, obtained by applying N excitation operators on a given a reference state $|\psi_0\rangle$:

$$|\phi\rangle = \prod_{i=1}^{N} e^{\theta_i T_i} |\psi_0\rangle , \tag{10}$$

where the $T_i = t_i - t_i^\dagger$ are a set of anti-Hermitian operators, the θ_i are free parameters, and the product is defined such that the terms with smaller values of the index i appear on the right, i.e., $\prod_{i=1}^{3} \hat{o}_i = \hat{o}_3\hat{o}_2\hat{o}_1$. Assuming $|\psi_0\rangle$ is normalized, the functional to be minimized is then

$$E_\phi = \langle\phi| \hat{H} |\phi\rangle . \tag{11}$$

The state $|\phi\rangle$ can be obtained without direct matrix exponentiation,[1, 2] and in this work we use the Scipy `expm_multiply` function to compute the action of a matrix exponential on a [3] vector, which is relatively efficient since the operator in each exponent in Supp. Eq. 10 is incredibly sparse.

In order to perform a gradient-based minimization, the derivative of the energy with respect to each parameter in the ansatz is needed. The analytical derivatives[4] then follow directly as

$$\begin{aligned}\frac{\partial E_\phi}{\partial \theta_i} &= \langle\phi| \hat{H} \prod_{j=i+1}^{N} \left(e^{\theta_j T_j}\right) T_i \prod_{k=1}^{i} \left(e^{\theta_k T_k}\right) |\psi_0\rangle \\ &\quad - \langle\psi_0| \prod_{k=i}^{1} \left(e^{-\theta_k T_k}\right) T_i \prod_{j=N}^{i+1} \left(e^{-\theta_j T_j}\right) \hat{H} |\phi\rangle ,\end{aligned} \tag{12}$$

where in the second line, we use the convention where products with indices starting at higher values correspond to a reverse-order product in which higher-index terms are on the right, i.e., $\prod_{j=3}^{1} \hat{o}_j = \hat{o}_1\hat{o}_2\hat{o}_3$. Recognizing that the second line is just the adjoint of the first, Supp. Eq. 12 can be simplified to

$$\frac{\partial E_\phi}{\partial \theta_i} = 2\Re \left(\langle\phi| \hat{H} \prod_{j=i+1}^{N} \left(e^{\theta_j T_j}\right) T_i \prod_{k=1}^{i} \left(e^{\theta_k T_k}\right) |\psi_0\rangle \right) . \tag{13}$$

In order to obtain a more compact expression for the ith parameter derivative, we partially expand the wavefunction expression in Supp. Eq. 10, grouping the unitaries up to and after i to define the notation for a state partially rotated with unitaries from 1 to i as $|\psi_{1,i}\rangle$:

$$|\phi\rangle = \left[\prod_{j=i+1}^{N} e^{\theta_j T_j} \right] \left[\prod_{k=1}^{i} e^{\theta_k T_k} \right] |\psi_0\rangle \tag{14}$$

$$= \left[\prod_{j=i+1}^{N} e^{\theta_j T_j} \right] |\psi_{1,i}\rangle \tag{15}$$

$$= |\psi_{1,N}\rangle . \tag{16}$$

Using this notation and defining $|\sigma_i\rangle = \prod_{j=N}^{i+1} \left(e^{-\theta_j T_j}\right) \hat{H} |\phi\rangle$ where $|\sigma_N\rangle = \hat{H} |\phi\rangle$, Supp. Eq. 13 becomes

$$\frac{\partial E_\phi}{\partial \theta_i} = 2\Re \langle\sigma_i| T_i |\psi_{1,i}\rangle . \tag{17}$$

While this analytical gradient formula implemented N times (one for each parameter) is already an improvement over its numerical counterpart in terms of precision, it can be defined recursively to provide a significant improvement for computational efficiency. The gradient algorithm proceeds by computing one parameter gradient using precomputed quantities from the previous parameter's gradient evaluation, significantly reducing CPU cost. We start the algorithm, the base case, at the left-most operator, $i = N$, where we need to calculate

$$\frac{\partial E_\phi}{\partial \theta_N} = 2\Re \langle\sigma_N| T_N |\psi_{1,N}\rangle . \tag{18}$$

At the second step, we need to compute

$$\frac{\partial E_\phi}{\partial \theta_{N-1}} = 2\Re \langle \sigma_{N-1}| T_{N-1} |\psi_{1,N-1}\rangle \,, \tag{19}$$

but this can be facilitated by using the fact that the new states $|\sigma_{N-1}\rangle$ and $|\psi_{1,N-1}\rangle$ needed at this stage can be obtained simply by applying a single matrix exponential to the states from the first step:

$$|\sigma_{N-1}\rangle = e^{-\theta_N T_N} |\sigma_N\rangle \,, \tag{20}$$

$$|\psi_{1,N-1}\rangle = e^{-\theta_N T_N} |\psi_{1,N}\rangle \,. \tag{21}$$

This continues to be the case at all later steps, where the states needed at each step can be similarly obtained from those at the previous step:

$$|\sigma_i\rangle = e^{-\theta_{i+1} T_{i+1}} |\sigma_{i+1}\rangle \,, \tag{22}$$

$$|\psi_{1,i}\rangle = e^{-\theta_{i+1} T_{i+1}} |\psi_{1,i+1}\rangle \,. \tag{23}$$

The benefit of this is that instead of applying all the Trotter operators in the ansatz for each single parameter, each parameter gradient only requires two `expm_multiply` calls and one sparse matrix vector multiplication. Stacking the $|\sigma_i\rangle$ and $|\psi_{1,i}\rangle$ into a two column matrix and computing the exponential action on that offers a numerical speedup in practice. As a consequence, obtaining the gradient with respect to all parameters is only roughly twice the cost of a single energy evaluation. Assuming the computational cost of other operations to be negligible, this recursive algorithm reduces the cost of computing a gradient from N^2 to $3N$. In the case of water in the STO-3G basis (140 operators) this represents a nearly 47-fold improvement. For a comparison, Table I lists the times required for a few VQE iterations of H_2O with the STO-3G basis set using both numerical and analytical gradients.

E. Energy discontinuities

As an adaptive algorithm, ADAPT-VQE self-consistently determines a quasi-optimal ansatz to describe the ground state of a particular Hamiltonian. However, as the nuclei move a new Hamiltonian is obtained. This means that any two distinct points on a potential energy surface will generally be treated with different ansatze. Not only will the two points generally have a different number of operators, but the order and composition of the ansatz will also generally differ. This means that the common shortcut in classical electronic structure theory which allows one to cancel systematic errors is not expected to work, and therefore, one must converge the ansatz growth tightly enough for the desired application.

As discussed in the text, two notable examples of this discontinuous behaviour occur for H_6. In order to better illustrate how these discontinuities are created, we plot the norm of the gradient vector as a function of the iteration (i.e., number of parameters). A separate convergence plot is shown for each of the molecules, LiH, BeH_2, and H_6 in Fig. 1.

While LiH and BeH_2 both exhibit rapid convergence, the strongly correlated H_6 has a distinctly different behavior. For short bond lengths (lighter colored lines) similar convergence behavior as the previous molecules is observed. In contrast, for longer bond lengths, a local minimum in the gradient norm begins to appear, requiring an increase in the number of parameters for tight convergence. This does not necessarily mean that the ansatz grown by ADAPT-VQE is not compact. H_6 is intrinsically more complicated, especially starting from a HF reference, and so it is expected to require more operators (and parameters). However, the consequence of this local minimum is that sometimes the algorithm may signal convergence prematurely, thus ignoring the extra operators that might be needed to make the ansatz sufficiently accurate. In Fig. 1(c), the difference between R = 2.4 and 2.5 Å involves a significant reduction in the number of parameters for ADAPT(ϵ_2), due to the fact that the 2.5 curve has a local minimum which crosses the convergence threshold before actual convergence is achieved. The 2.4 Å local minimum is less deep, and doesn't cross the threshold. As such, the algorithm needs to climb out of the local minimum to find the "true" convergence point where the gradient finally drops below the threshold with a larger number of parameters. The consequence of this is the significant drop in the number of parameters (and accuracy) going from 2.4 to 2.5 Å. Future work will focus on analyzing difficult cases such as these to determine better convergence criteria that are less susceptible to local minimum convergence.

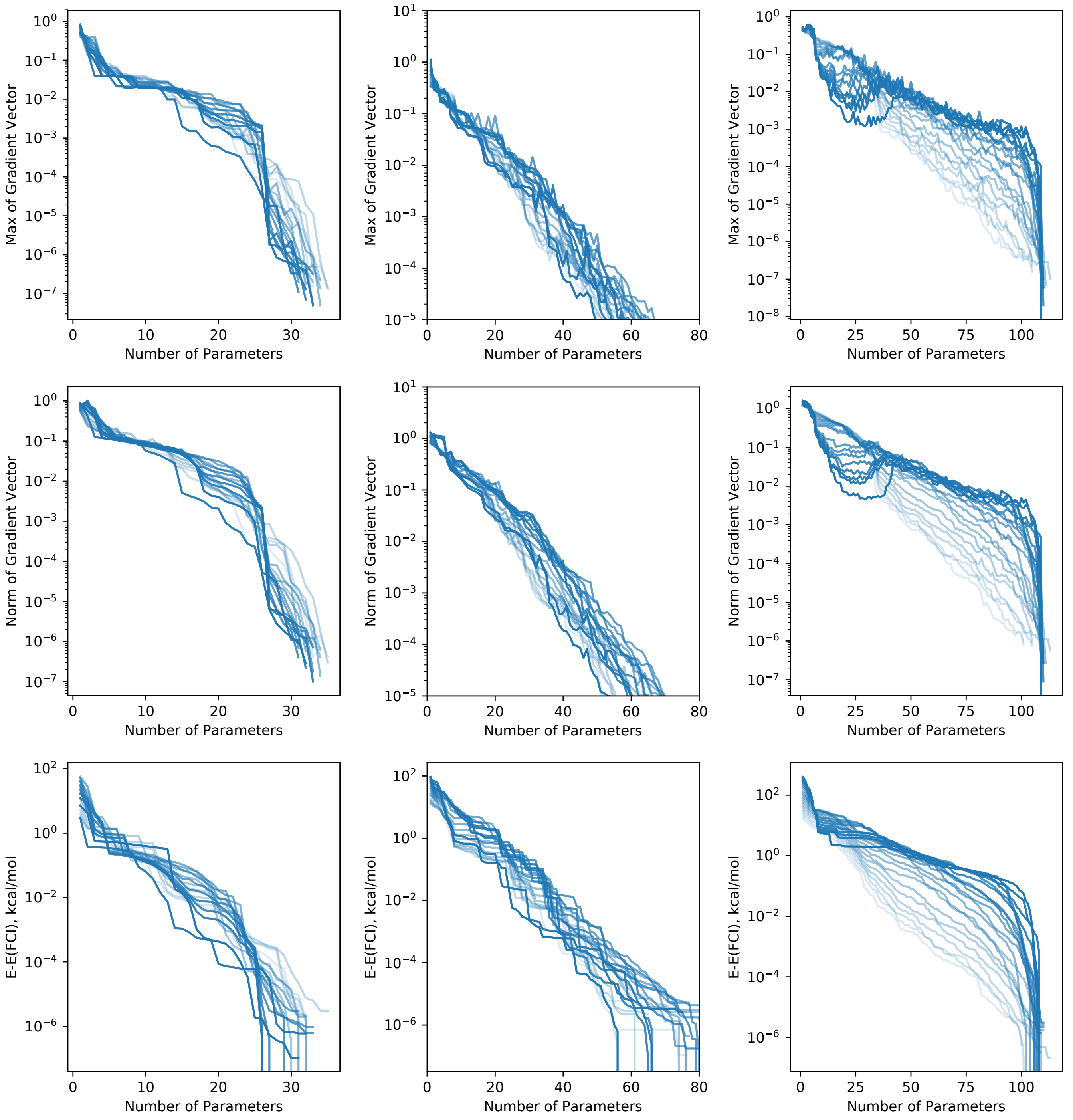

(a) LiH

(b) BeH_2

(c) H_6

Supplementary Figure 1: Convergence of the gradient max (top), gradient norm (middle), and energy error (bottom) at different bond lengths for (a) LiH, (b) BeH_2, and (c) H_6. Each line corresponds to a distinct bond distance, ranging from light to dark as the distance increases. For both LiH ($R_{eq} = 2.39$ Å) and BeH_2 ($R_{eq} = 1.342$ Å), the bond distance ranges from 0.5 R_{eq} - 2.5 R_{eq}. For H_6, the bond distance ranges from 0.5 Å- 2.5 Å.

Supplementary References

[1] Higham, N. J. & Al-Mohy, A. H. Computing matrix functions. Acta Numerica **19**, 159–208 (2010). URL http://www.journals.cambridge.org/abstract{_}S0962492910000036.

[2] Al-Mohy, A. H. & Higham, N. J. Computing the Action of the Matrix Exponential, with an Application to Exponential Integrators. SIAM Journal on Scientific Computing **33**, 488–511 (2011). URL http://epubs.siam.org/doi/10.1137/100788860.

[3] Jones, E., Travis, O. & Peterson, P. SciPy: Open source scientific tools for Python (2001–). URL http://www.scipy.org/.

[4] Romero, J. et al. Strategies for quantum computing molecular energies using the unitary coupled cluster ansatz. Quantum Science and Technology **4**, 014008 (2018). URL http://stacks.iop.org/2058-9565/4/i=1/a=014008?key=crossref.0e74114499ad0be8c7997e37f733ca0d.
